# Achieving High Performance in AC-Field Driven Organic Light Sources

**DOI:** 10.1038/srep24116

**Published:** 2016-04-11

**Authors:** Junwei Xu, David L. Carroll, Gregory M. Smith, Chaochao Dun, Yue Cui

**Affiliations:** 1 Center for Nanotechnology and Molecular Materials, and Department of Physics, Wake Forest University, Winston-Salem, NC 27109, USA; 2 Key Laboratory of Luminescence and Optical Information (Ministry of Education), Institute of Optoelectronics Technology, Beijing Jiaotong University, Beijing 100044, P.R. China

## Abstract

Charge balance in organic light emitting structures is essential to simultaneously achieving high brightness and high efficiency. In DC-driven organic light emitting devices (OLEDs), this is relatively straight forward. However, in the newly emerging, capacitive, field-activated AC-driven organic devices, charge balance can be a challenge. In this work we introduce the concept of gating the compensation charge in AC-driven organic devices and demonstrate that this can result in exceptional increases in device performance. To do this we replace the insulator layer in a typical field-activated organic light emitting device with a nanostructured, wide band gap semiconductor layer. This layer acts as a gate between the emitter layer and the voltage contact. Time resolved device characterization shows that, at high-frequencies (over 40 kHz), the semiconductor layer allows for charge accumulation in the forward bias, light generating part of the AC cycle and charge compensation in the negative, quiescent part of the AC cycle. Such gated AC organic devices can achieve a non-output coupled luminance of 25,900 cd/m^2^ with power efficiencies that exceed both the insulator-based AC devices and OLEDs using the same emitters. This work clearly demonstrates that by realizing balanced management of charge, AC-driven organic light emitting devices may well be able to rival today’s OLEDs in performance.

Carrier drift under uniform external electric field is the dominate mechanism of free carrier injection and transportation in organic light-emitting diodes (OLEDs)^1^,^2^,^3^. Electrons and holes are injected as polarons from highly conductive, metallic, cathodes and anodes. They are then transported through the highest occupied molecular orbital (HOMO) and lowest unoccupied molecular orbital (LUMO) levels of organic semiconductors to a light emission layer respectively, resulting in the formation of excitons and radiative recombination transitions^4^,^5^. Thus, it is crucial to reduce barrier heights for the carrier injection at the metal/organic and organic/organic interfaces^6^,^7^,^8^,^9^,^10^. For the same reason, heat dissipation at injection barriers interfaces is not trivial for energy loss in OLEDs^11^. Hot carriers exceed the energy gap of the emitting material, so the excess energy is wasted thermally, giving rise to a lower power efficiency. The reduction of the carrier injection barriers by decreasing the difference between adjacent energy levels is now a common method to optimize the efficiency of OLEDs^7^,^12^,^13^,^14^,^15^.

However, recently many researchers have been drawn to understanding alternating current driven, organic electroluminescent devices (AC-OEL) which avoid losses at interfaces by the use of an AC field to create polarization currents in the active organic layer for light emission^16^,^17^,^18^,^19^,^20^,^21^,^22^,^23^,^24^,^25^. In these devices, power loss is limited to dielectric losses and efficiency and luminance of the AC-OEL have been attributed to the cross-section for the creation of free carrier by the field. Thus they are said to be “field-activated”.

For most defect free, light emitting, semiconducting polymers, the cross-section for the field generation of a polaron or exciton is small and with the massive reduction of injected carriers from electrodes this means the emitting layer will need to be doped. This is can be done blending dopants into the emitter or using a hole or electron generation layer as a carrier source^16^,^19^,^20^,^26^. Normal hole generation layers (HGLs) include N,N,N′,N′-tetrakis(4-Methoxy-phenyl)benzidine (MeO-TPD): tetrafluoro-tetracyanoquinodimethane (F4TCNQ)^16^, Poly(3-hexylthiophene) (P3HT):F4TCNQ^19^,and 4,4′,4″-Tris(N-3-methylphenyl-N-phenylamino)triphenylamine (m-MTDATA):F4TCNQ^27^. Bathophenanthroline (BPhen):Cs^16^, Cs_2_CO_3_:BPhen^28^, and 2,2′,2″-(1,3,5-Benzinetriyl)-tris(1-phenyl-1-H-benzimidazole) (TPBi):Cs2CO_3_^29^ are good choices for electron generation electron-donor/electron-acceptor (D/A) system. But, in order to prevent hot carrier direct injection into the device, single or double dielectric layers (in asymmetric or symmetric structures) should be utilized generally^30^. Unfortunately, the high dielectric constant of such materials (HfO_2_^16^, P(VDF-TrFE-CFE)^31^, SiO_2_^25^, and LiF^28^), means that a nontrivial applied voltage drop on the dielectric layer, rather than functional organic layers, results and high driving voltages are necessary for the insulating and capacitive AC-OEL. Moreover, dielectric losses can be large. Most importantly, however, the insulating nature of this layer means that there is little charge compensation allowed in the device and a space charge can accumulate during operation at high brightness.

In this work, we demonstrate that n-type ZnO, a typical wide band gap (~3.2 eV) semiconductor, can be used as an ideal substitution of high-k dielectric layer in AC-OEL devices, serving the function of a “passive carrier management gate”. By utilizing a ZnO nanoparticle (NPs) layer between anode and HGL not only hot holes are prevented from direction injection in the negative half of AC cycle, but electron extraction in HGL [Poly(4-butylphenyl-diphenyl-amine) (Poly-TPD):F4TCNQ] is facilitated. As we show, this essentially balances the charge in the device. This function of the ZnO gate is demonstrated in the Poly-TPD:F4TCNQ heterojunction system for this work, but may be applied generally.

## Results and Discussion

**Figure 1:**
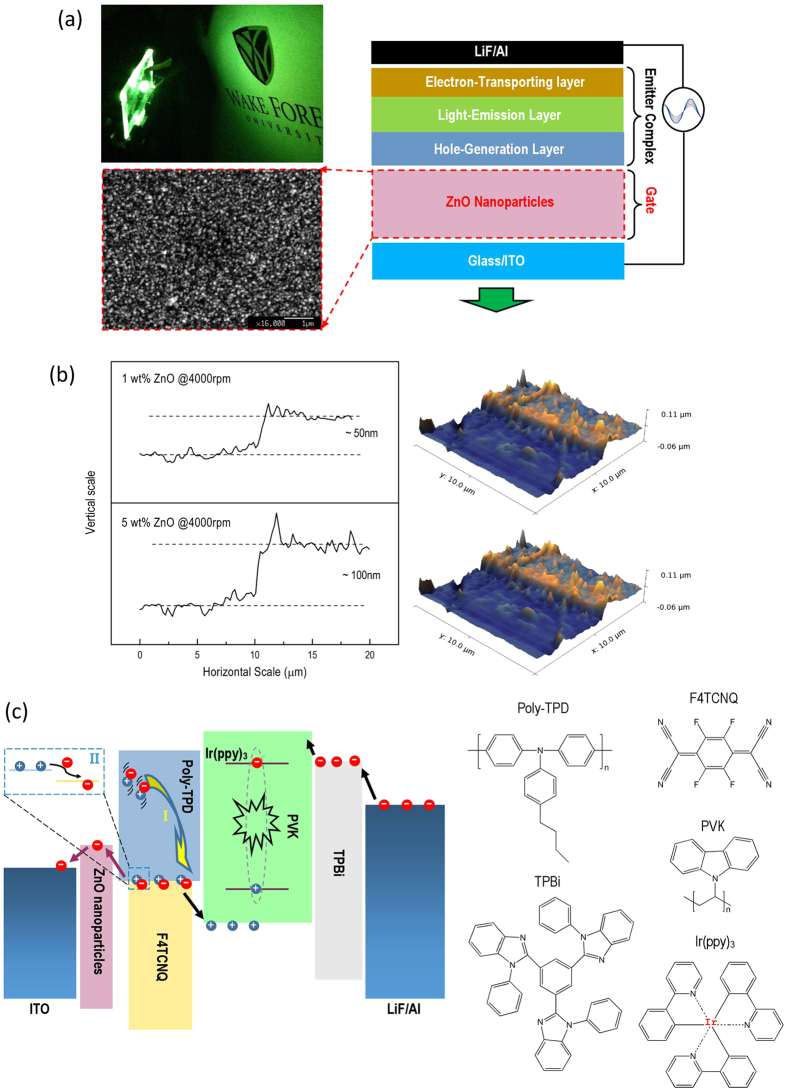
The structure of alternating current driven, organic electroluminescent (AC-OEL) devices with ZnO gate layer. (**a**) The high-brightness AC-OEL device configuration consists of ZnO gate and organic emitter complex. The photo of AC-OEL device has a luminance over 10,000 cd/m^2^. SEM image of ZnO NPs shows the morphology of gate layer. (**b**) The thicknesses of ZnO gate layers spun coat by different concentrations of ZnO NPs solution (1 wt% and 5 wt%). (**c**)The energy level diagram of the AC-OEL devices and organic molecule structures employed in this work.

The specific configuration of the AC-OEL used in this work is shown in Fig. 1(a), with the three-layer emitter complex, the gate structure, and the electrodes marked separately. To fabricate the structure we begin with spin-coating a layer of ZnO NPs (SEM image is shown Fig. 1(a)) onto the 2.5 cm × 2.5 cm glass substrate which has been pre-coated with 100 nm of ITO. This will function as a gate for carrier management. AFM images typical of 50 nm and 100 nm gate layers spun cast using 1 wt% and 5 wt% ZnO NP solutions in solvent, are shown in Fig. 1(b). The XRD of ZnO NPs layers used in the work are given as well in Supporting Information (SI) 1.

Poly-TPD doped with 10 wt% F4TCNQ was used as HGL and spun cast to a thickness of 70 nm on top of the ZnO layer (using a concentration of 10 mg/mL in chlorobenzene). This layer will function as the main source of holes in our devices.

A guest-host system was used in the light-emission layer (EML). This 150 nm layer contained the highly efficient metallo-organic phosphores, tris[2-phe-nylpyridinato-C 2, N ]iridium(III) [Ir(ppy)_3_], and a well-known p-type host polymer, Poly (N-vinylcarba- zole) (PVK). Thus, both singlet and triplet excitons could be harvested during device operation.

Finally, a 30 nm electron transport layer (ETL) of TPBi was spun cast in a glove box and this was then transferred into a high vacuum (~2 × 10^−7^ Torr) evaporator where a LiF/Al electrode was deposited. To be clear, we show the molecular structures of the functional organic materials employed in the work in Fig. 1(c).

We note here that while the overall construction seems similar to those seen in insulating AC organic EL devices^16^,^24^ where the HGL and ETL are effectively charge sources activated through a Zener breakdown process with applied field^32^, in this system the insulators have been replaced by a single gate structure and the HGL and ETL layers must be chosen specifically to allow barrier formation at the interfaces.

To fully understand the mechanisms of charge generation in D/A system (Poly-TPD:F4TCNQ), we must first consider the two sources of holes as shown in Fig. 1(c).

### Source I (Polarization current)

The first charge generation process is associated with the polarization current which normally exists in materials under an applied time-varying electric field. Molecules are being polarized under the influence of the electric field and displaced positive and negative charges are accumulating on sites throughout the volume randomly, as a bound charge density. These electron-hole pairs can become unbound in sufficiently large field assuming there is a local defect to assure momentum conservation. Alternatively, the bound pair can migrate in the field to the Poly-TPD/F4TCNQ interfaces^33^. At the interface, these electron-hole pairs may form charge transfer states that dissociate to mobile carriers^34^. Since the electric field varies with time, then the charge displacement is time dependent. The displacement yields a polarization current which is proportional to the time derivative of the field 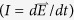
35.

### Source II (Electron-donor/electron-acceptor heterojunctions)

Poly-TPD and F4TCNQ are known as a strong electron donor and accepter respectively. When 10% F4TNQ is doped in Poly-TPD, electrons can easily tunnel from HOMO states of electron donor (Poly-TPD) to the LUMO states of electron acceptor (F4TCNQ) through a narrow depletion zone^36^. This suggests that an electron-hole pair generated in doped organic p-n heterojunctions, dissociates under intense electric field. The electric-field-assisted bipolar charges spout from the internal charge separation^37^,^38^.

The combination of these two sources of charge generation is responsible for the electron-hole pair population in the HGL. The unstable electron-hole pairs are easily dissociated into free holes in the HOMO state of Poly-TPD and electrons in the LUMO state of F4TCNQ at the interface of Poly-TPD/F4TCNQ. Under external electric field, free holes drift into EML for light emission.

**Figure 2:**
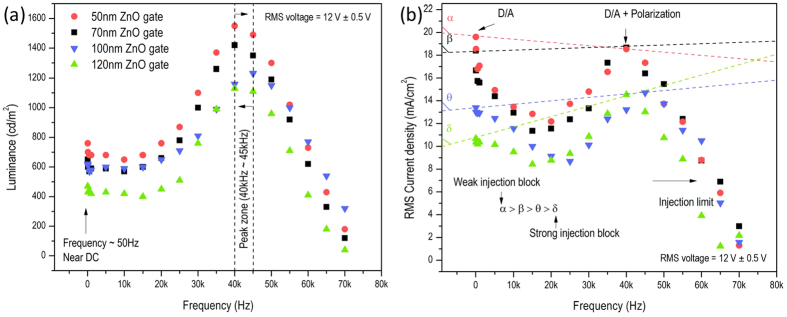
Performance of phosphorescent green AC-OEL devices with various thicknesses of ZnO gate. (a) The luminance as function of driving frequency (from 50 Hz to 70 kHz). (**b**) The RMS current density as function of driving frequency (from 50 Hz to 70 kHz).

The gate structure, however, changes the balance of these charge sources according to frequency and applied voltage. The frequency-dependent characteristics of AC-OEL devices with ZnO gates of different thicknesses (50 nm, 70 nm, 100 nm, and 120 nm) are shown in Fig. 2(a,b). There is a noticeable difference in the AC-OEL devices’ current and emissive response with frequency as the gate thickness is increased. For instance, Fig. 2(b) shows a strong thickness-dependence in carrier injection (current) in the low frequency range (below 500 Hz). Near DC frequencies (<50 Hz), the AC-OEL devices with 50 nm, 70 nm, 100 nm, and 120 nm gates all show relatively low levels of light generation (470 cd/m^2^, 620 cd/m^2^, 650 cd/m^2^, and 760 cd/m^2^) at current densities of 10.4 mA/cm^2^, 13.0 mA/cm^2^, 16.7 mA/cm^2^, and 18.5 mA/cm^2^, respectively. However, these luminances increase dramatically at 40 kHz for instance (1,130 cd/m^2^, 1,230 cd/m^2^, 1,420 cd/m^2^, and 1,550 cd/m^2^), nearly twice that of low frequency driving, while the current densities of the devices remain at the nearly same level (18.7 mA/cm^2^, 18.5 mA/cm^2^, 14.5 mA/cm^2^, and 14.7 mA/cm^2^, respectively). Thus, it is reasonable to suggest that the current densities at very low and DC frequencies mainly depend on the contribution of external hot carrier injection, rather than carrier generation within the devices. This is made clearer in Fig. 2(b), α, β, θ, and δ are slopes of lines connecting the data points between at 50 Hz and the data points locating at luminance peaks at high frequency. The larger the slope the higher the injection barrier for free carriers near DC. We attribute this graphical result: α > β > θ > δ, to the fact that the voltage drop over ZnO layer dramatically increases. Note that the dielectric constant (~3) of the organic multi-layer is incompatible with that of the inorganic semiconductor gate layer (~8). So, the tunneling probability of the positive charges through gate is greatly suppressed. This gives a lower current density (for near DC frequencies) with the thicker ZnO gates. Thus, we reason that carrier injection is the dominant mechanism of free charge in these ZnO gated AC-OEL devices at low frequencies and the behavior is similar to that of an AC driven OLED^29^.

Conversely, the high-frequency electric field (where the field is held constant RMS magnitude) induces a large population of the free carriers due to the contributions of charge injection and polarization. The luminance and current peaks in the frequency sweep are the direct evidence to show this point.

**Figure 3:**
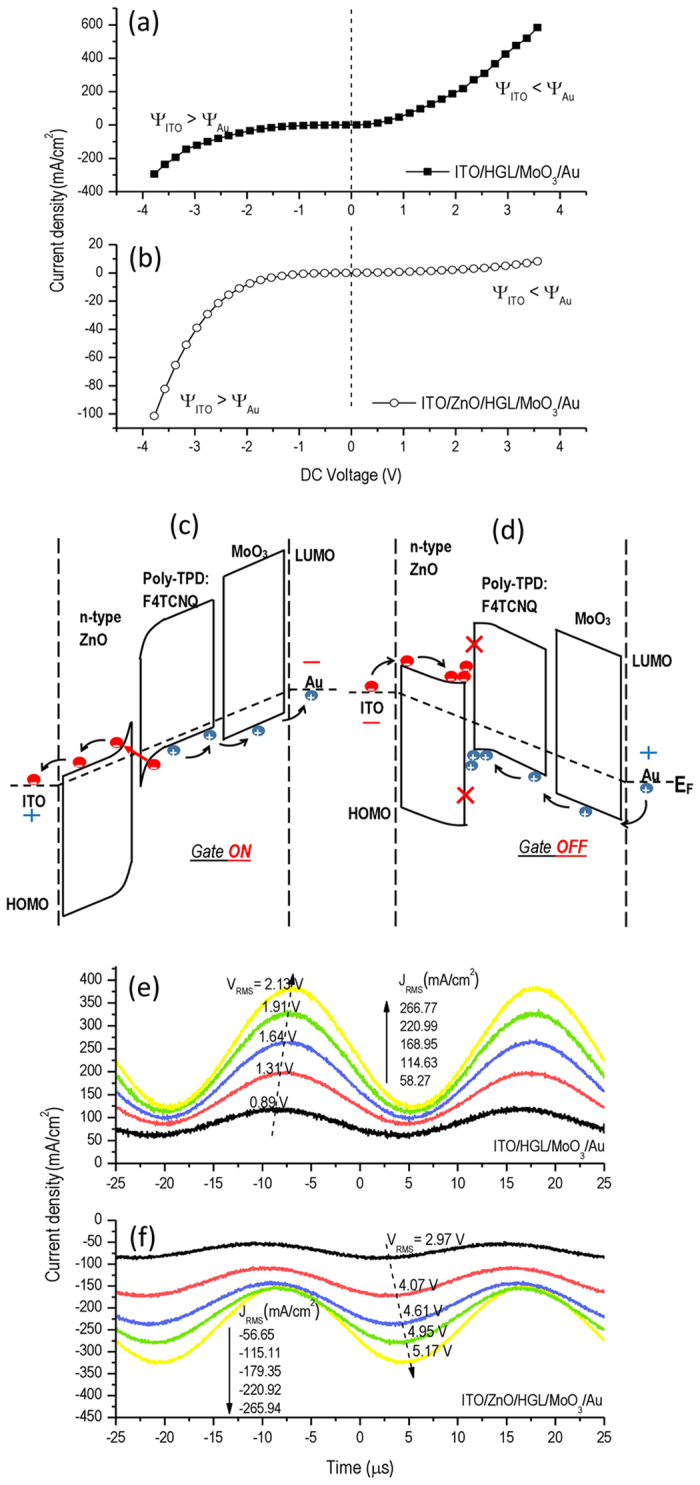
Analysis of the functionality of ZnO gate in hole-only devices in the positive and negative bias of AC voltage cycles. (**a,b**) Current density versus voltage characteristic under DC driving in the devices of ITO (100 nm)/ZnO (~100 nm)/Poly-TPD:F4TCNQ (~70 nm)/MoO3 (15 nm)/Au (100 nm) and ITO (100 nm)/ZnO (~100 nm)/MoO3 (15 nm)/Au (100 nm). (**c,d**) Energy band diagrams of AC-OEL devices in forward and reverse bias. (**e,f**) Time response of cell current in ITO (100 nm)/ZnO (~100 nm)/Poly-TPD:F4TCNQ (~70 nm)/Au (100 nm) and ITO (100 nm)/ZnO (~100 nm)/Au (100 nm).

To further investigate the carrier manipulation of semiconductor gate, the hole-only devices with a ZnO gate were studied using the structure: ITO/ZnO NPs(~100 nm)/Poly-TPD: F4TCNQ(~70 nm)/MoO_3_(15 nm)/Au(100 nm) and ITO/Poly-TPD: F4TCNQ(~70 nm)/MoO_3_(15 nm)/Au(100 nm). Figure 3(a,b) show the J_RMS_ as a function of V_RMS_ for devices with and without ZnO gate layer under the forward and reverse DC bias. In the absence of ZnO gate, the device exhibits nearly symmetric curve (584.6 mA/cm^2^ at 3.57 V versus -293.87 mA/cm^2^ at −3.77 V). This is because the symmetric energy barriers at the interface (~0.3 eV) of ITO and HGL and that of HGL and Au (~0.1 eV) for carrier injection. However, the device with the ZnO semiconductor shows a “gating” effect in the I-V characteristics (8.2 mA/cm^2^ at 3.67 V versus −101.5 mA/cm^2^ at −3.78 V). We illustrate this using a band alignment diagram of the hole-only devices in Fig. 3(c,d). In the forward cycles, the direction of external electric field points from ITO towards Au. The high density of positive charges in the HOMO state of Poly-TPD are strongly drifted to MoO_3_ then to Au. On the other hand, the excess electrons trapped in HGL are transferred by the strong electric field and encounter the energy barrier at HGL/ZnO interface, then tunnel to the CB of ZnO. The electron extraction in HGL dramatically facilitates the electron hopping rate from Poly-TPD to F4TCNQ, resulting in a larger population of positive charge. In other words, the ZnO gate “opens the door” of electron extraction and hole regeneration in HGL. As the applied AC voltage switches to reversed fields in the power cycle, the electric field tilts the Fermi level. Holes in the LUMO of Poly-TPD are driven towards the opposite direction and are accumulated at the interface of ZnO and HGL because of high tunneling barrier (~2.2 eV). The ZnO gate “shuts the door” for electron extraction before the next forward cycle arrives. A high electron injection barrier (~2.5 eV) is observed as well. The accumulated carriers trapped in HGL will be moved or neutralized in the next cycle of charge regeneration. Therefore, the ZnO layer plays a role of electron extraction layer (“gate on”) in the forward bias and works as dielectric layer (“gate off”) in the reverse cycle.

Figure 3(e,f) shows the time-resolved current characteristics under AC driving. Waveforms of current density indicate not only increased amplitude with rising RMS driving voltage but are also positive (without ZnO) or negative (with ZnO). More importantly, the negative current density in the device with ZnO gate suggests that the free electrons are transferred from Au side to ITO side. In the perspective of AC driven devices, the ZnO gate facilitates or “pumps” the excess electrons traped in HGL, resulting in a high population of positive charge.

**Figure 4:**
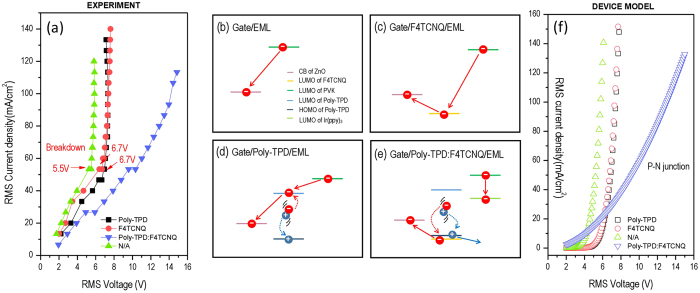
I-V performance and theoretical models of the AC-OEL devices with various hole generation layers (HGL). (**a**) Experimental RMS current density as function of RMS voltage utilizing Poly-TPD:F4TCNQ, Poly TPD, F4TCNQ as HGL or absence, respectively. (**b–e**) Are electronic band structures analysis with various HGLs. (**f**) Theoretical RMS current density as function of RMS voltage by fitting in tunneling model and P-N junction model.

To further validate the electron extractor and hole blocker function of the ZnO gate, under intense high-frequency electric field in the gated AC-OEL devices, the Poly-TPD:F4TCNQ (doped HGL) was substituted by pure Poly-TPD or F4TCNQ in AC-OEL devices or absence. The experimental J_RMS_-V_RMS_ curves of these devices are shown in Fig. 4(a). In the AC-OLED device, without the electron blocking layer, electrons are injected from cathode, then transferred to the anode without recombination, which causes a poor EL performance (shown in SI 4) and a low breakdown voltage (5.5 V). This, we suggest, is explained by the fact that the device missing HGL layer, establishes an electron passage through the LUMOs of functional organic layers to CB of ZnO (shown in Fig. 4(b)).

Meanwhile, the same tunneling behavior was observed when a layer of F4TCNQ was sandwiched by ZnO and PVK:Ir(ppy)_3_. The J_RMS_ of the device increases rapidly as function of V_RMS_ because electrons injection from Ir(ppy)_3_ can be drifted to ZnO NPs through F4TCNQ molecules which is an excellent electron acceptor (shown in Fig. 4(c)). An identical curve was found for pure Poly-TPD since free electrons dissociated from the electron-hole pairs due to AC electric field have to stay in the LUMO state of Poly-TPD (shown in Fig. 4(d)), rather than be transferred to the HOMO states of the electron acceptor (F4TCNQ) in D/A system (shown in Fig. 4(e)). The high density of electrons in Poly-TPD enormously increases the possibility of electron breakdown in the device. A turning point in J–V curve can be observed and confirms the fact that the device is breaking down at a voltage of 6.7 V. In contrast, in the Poly-TPD:F4TCNQ device, current density increases without breakdown while applied AC voltage increases since free charges are well-managed at the ZnO/HGL interface. The electron-hole pairs are dissociated into free electrons and holes staying in the HOMO state of F4TCNQ and the LUMO state of Poly-TPD, respectively, illustrated in Fig. 4(e). In the forward bias of AC cycles, the injected electrons in PVK will not break through the barrier to reach ZnO, but instead, hop to the LUMO state of Ir(ppy)_3_ for exciton recombination.

We use a previously established tunneling model^39^,^40^ coupled with a P-N junction model^41^ to further interpret the experimental data. Modeling details can be found in the supporting information. The modeling results of the J_RMS_–V_RMS_ characteristics of the devices are in excellent agreement with experimental measurements in Fig. 4(f). Together with the experimental demonstration, this suggests that the charge generation mechanism due to field polarization indeed has great impact on majority of free hole carrier population, and that the ZnO gate has a non-trivial influence on carrier extraction and balancing of the dual carrier injection and transport under an AC electric field.

So far, we have demonstrated the electronic functionality of the ZnO semiconductor “gate” in AC-OEL devices. Specifically, the ZnO gate is responsible for carrier management in the Poly-TPD:F4TCNQ by facilitating (gate on) and blocking (gate off) the electron transport passage in different halves of AC power cycle. Furthermore, it is worth comparing different gates, 100 nm ZnO, 100 nm P(VDF-TrFE-CFE) in a same AC-OEL devices configuration, and AC-OLED directly.

**Figure 5:**
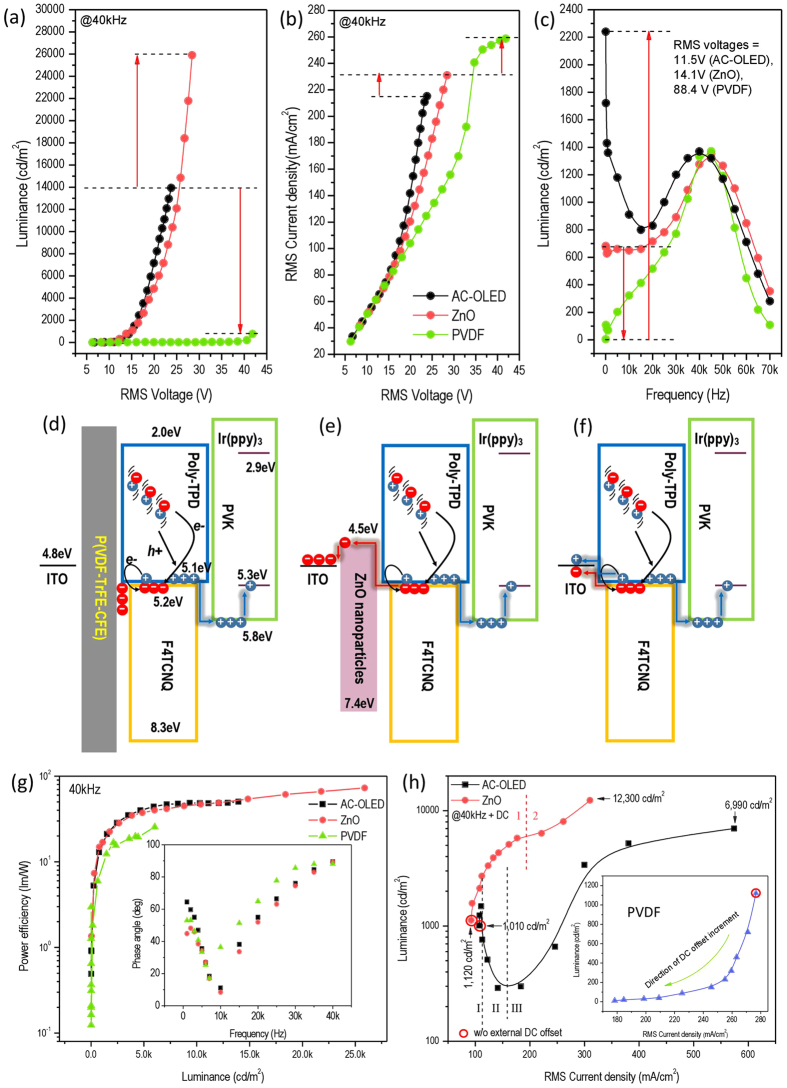
High-frequency (40 kHz) performance of the AC-OEL devices with 100 nm ZnO gate and 100 nm P(VDF-TrFE-CFE) insulator, and AC-OLED. (**a**) RMS current density as function of RMS voltage. (**b**) Luminance as function of RMS voltage. (**c**) Luminance as function of frequency. (**d**–**f**) The carrier transportation mechanisms of AC-OEL devices with ZnO gate and P(VDF-TrFE-CFE) insulator, and AC-OLED. (**g**) Power efficiency plot as function of luminance. Inset shows the phase angle characteristics. (**h**) Luminance versus RMS current density characteristics of the AC-OEL devices with ZnO gate and P(VDF-TrFE-CFE) insulator, and AC-OLED while an increasing DC voltage offset are added to AC driving voltage. 7.1 V and 4.2 V of DC voltages are added to AC voltages when AC-OLED and ZnO device reach the maximum luminance respectively.

First, the “turn-on” voltage: in Fig. 5(a), the turn-on voltage at 40 kHz of the device with ZnO gate is 10.5 V (RMS value) which is much lower than that (29.1 V) of the device with P(VDF-TRFE-CFE) insulator. However, turn-on voltages of the devices with the ZnO gate (10.5 V) and without (9.0 V) are nearly identical.

Second, the maximum brightness: Fig. 5(a) shows that the peak brightness at 40 kHz of the AC-OEL device with the ZnO gate is as high as 25,900 cd/m^2 ^at 28.4 V while the P(VDF-TRFE-CFE) device has a luminance of 770 cd/m^2^ at 41.9 V (maximum brightness 6,030 cd/m^2^ at 108.9 V is not indicated in the plot). As a reminder, we state that these devices are built on glass without the aid of output coupling and output was measured using a fiber optic light probe. Thus light captured by the glass substrate is not accounted for. The devices without gate or insulator exhibit a maximum luminance of 13,940 cd/m^2^ at 23.8 V. However, the current characteristics in Fig. 5(b) show the opposite trend; that the devices with ZnO gate, P(VDF-TRFE-CFE) insulator, and AC-OLED have current densities of 231.0 mA/cm^2^, 258.7 mA/cm^2^, and 215.2 mA/cm^2^, respectively. This suggests the carrier transport within the devices have become unbalanced shifting the recombination zone away from the emitter layer.

Third, the low frequency response: In Fig. 5(c), we observed that the luminance is greatly suppressed to 10 cd/m^2^ at 50 Hz by the P(VDF-TRFE-CFE) insulator, whereas, AC-driven OLED shows a much higher luminance of 2,240 cd/m^2^ due to the dramatically enhanced carrier injection. As would be anticipated, a luminance of 680 cd/m^2^ is obtained in the device with ZnO gate, which demonstrates the gate function of ZnO as distinguished from the insulator or direct injection devices.

This suggests that the significant optical and electric differences of the various charge injection responses are due to the charge transportation properties and band gap structures of semiconductor and insulator. The dielectric constant of P(VDF-TrFE-CFE), as high as 40 at 40 kHz^42^, means that the carrier injection is limited and the applied electric field is not enough to effectively generate carriers within the emitter. There is enormous free charge accumulated at the interface of the P(VDF-TrFE-CFE) and HGL in Fig. 5(d). In the case of ZnO, the gate facilitates electron extraction from HGL (Poly-TPD:F4TCNQ) which greatly enhances the generation rate of positive charge. Electron extraction over ZnO and hole regeneration in Poly-TPD:F4TCNQ tremendously promotes carrier injection in positive half of AC power cycle at low voltage (see in Fig. 5(e)). In the reversed bias, the hole carriers are trapped at the ZnO/HGL interface resulting in a lower current density in Fig. 5(f) compared with the devices that have no gate or utilize an insulator. The theoretical analysis on carrier injection and transportation is confirmed by power efficiency in Fig. 5(g). The 40 kHz driven lighting device with ZnO gate exhibits the highest efficiency of 72.9 lm/W, compared 25.4 lm/W with P(VDF-TrFE-CFE) and 50.3 lm/W in AC-drive OLED. Table 1 summaries the performances of AC-OEL devices with different gates at high frequency (40 kHz) or low frequency (50 Hz).With comparison to the power efficiency as low as to 2 lm/W at 50 Hz, we conclude that polarization current contribution facilitates more efficient carriers injection than the direct injection of hot carriers, which exhibits dramatically high power efficiency at 40 kHz.

The crucial condition for the ZnO gate to be “open” or “closed” is band alignment of the AC-OEL devices and the direction of net electric field applied to the device. Thus, a reasonable external DC electric field can promote the electron extraction from HGL to ZnO, resulting in a higher density of hole carriers. Conversely, the direct current injection can be significantly suppressed due to the high k insulator. So it is not hard to understand the inset in Fig. 5(h), which shows the luminance - V_RMS_ curve where a DC offset has been added to the device during AC driving.

However, the analysis of the luminance - V_RMS_ characteristics comparison between ZnO gated and AC-OLED in Fig. 5(h) is more complicated. This is because the luminance - V_RMS_ curves are divided into parts (zone “1” and “2” for ZnO gate; zone “I”, “II”, and “III” for AC-OLED). For the device with ZnO gate, an external DC electric field accelerates the rate of electron extraction over the ZnO and establishes hole accumulation, leading to the increasing brightness (zone “1”). In the device without gate, the DC electric field promotes electron extraction as well as hole extraction, which continuously increases current density (shown in SI 6). However, the luminance of the AC-OLED drops rapidly after a small bump (between zone “I” and “II”) because the positive external DC offset trades off against the slight negative offset in AC waveforms in the first stage, resulting in a reduced total voltage. When the external DC electric field is nontrivial compared to AC field, both of the devices (with and without ZnO gate) work as traditional OLEDs and show the peak luminances of 12,300 cd/m^2 ^at 309.3 mA/cm^2^ and 6,990 cd/m^2^ at 575.8 mA/cm^2^ (zone “2” and “III”), respectively.

In summary, we propose that, due to the gating effect at the interface between n-type and p-type semiconductors, ZnO is an ideal gate material to replace traditional dielectric layers in the AC-driven EL devices. Based on general band alignment in AC-OEL devices, the energy band diagrams at the interface of gate and HGL in the forward and reverse cycles of AC voltage are given and demonstrated in hole-only device experiments under positive and negative bias. Four distinct types of devices with different HGLs (F4TCNQ, Poly-TPD, Poly-TPD: F4TCNQ, or absence) were fabricated to confirm the validity of ZnO gate function and hole generation mechanism due to polarization in the experiment and modeling simulation. A comparison between AC-OLED and AC-OEL devices with ZnO and P(VDF-TrFE-CFE) reveals distinct trends in the performance of luminance, frequency-dependent, and DC-offset characteristics. From the viewpoint of the broader electroluminescence community, inorganic semiconductor gates provide a novel strategy to utilize free charge injection as well as polarization current in organic thin film EL devices for the development of next generation, high brightness, high power efficiency, AC-DC hybrid electroluminescence devices.

## Methods

### Materials and device fabrication

All devices were fabricated on a glass substrate with a pre-coated 100 nm ITO film with a sheet resistance of approximately 10 Ω. The substrates were cleaned in an ultrasonic bath with acetone followed by methanol and isopropanol for 1 hour each. The ITO substrates subsequently were dried in a vacuum oven for 2 hours and treated with UV-ozone for 15 min. ZnO NPs aqueous solution (50 wt%, average diameter ~35 nm, Aldrich) was purchased and diluted to 1 wt% and 5 wt%. The ZnO gate layer was spun coat with 1 wt% and 5 wt% ZnO NPs aqueous solution (50 wt%, average diameter ~35 nm, Aldrich). Then, a doped hole generation layer of doped Poly(4-butylphenyl-diphenyl-amine) (Poly-TPD)/ 2,3,5,6-Tetrafluoro-7,7,8,8-tetracyanoquinodimethane (F4TCNQ) is fabricated at 2500 rpm in a weight ratio of 10:1. The emission layer consisted of a blend of poly(N-vinylcarbazole) (PVK) as a co-host and 10 wt% fac-tris(2- phenylpyridinato)iridium(III) (Ir(ppy)3) as the dopant. The emitting layers were obtained by spin coating the 18 mg/mL PVK:Ir(ppy)3 blend in chlorobenzene at 3000 rpm. As an electron injection layer, 1 and 3, 5-tris (2-N-phenylbenzimidazolyl) benzene (TPBi) is dissolved in a mixture of formic acid and water (5:1) and spin cast at 3500 rpm before moving to a heated vacuum oven for 30 min. Then, a 1 nm layer of LiF and a 100 nm layer of Al was deposited as electrode in high vacuum. The active area of the AC-OEL pixels was 15.0 mm^2^.

### Device electroluminescence measurement

The AC driven field-induced polymer EL devices in this report are measured in ambient air at atmospheric pressure and room temperature (25 °C) without sealing. A 200 MHz function/arbitrary waveform generator (Agilent 33220A) connected to an amplifier (Trek PZD700A M/S) provides a sinusoidal signal with suitable voltage and frequency. A power analyzer (Zimmer LMG95) is utilized to read the root mean square (RMS) value of voltage and current on the AC-OEL devices. At the same time, voltage waveforms and current waveforms are recorded from a Tektronix TPS 2024B oscilloscope to validate the voltage and current values. A photometer (ILT 1400-A) is used to measure the out-coupling luminance. The entire system is connected and controlled by a computer (shown in SI 7). In order to maintain accurate and reliable measurements of luminance and efficiency, each turn-on measurement of the pixels was integrated over 2000 ms and averaged 5 times instead of fast sweeping for good-looking curves.

### SEM and AFM images

A scanning electron microscope (SEM, JEOL 6330) was utilized to measure the morphology of ZnO NPs gate. The thicknesses of ZnO gates were calibrated by an atom force microscope (AFM, Asylum Research) and a surface profiler (Alpha-Step 500).

## Additional Information

**How to cite this article**: Xu, J. *et al.* Achieving High Performance in AC-Field Driven Organic Light Sources. *Sci. Rep.* **6**, 24116; doi: 10.1038/srep24116 (2016).

## Supplementary Material

Supplementary Information

## Figures and Tables

**Table 1:** Summary of the performance of AC-OEL with different gates at high frequency and low frequency.

Gate material	High frequency (at 40 kHz)			Low frequency (at 50 Hz)		
	Turn-on voltage [V]	Max. brightness [cd/m^2^]	Max. Power eff. [lm/W]	Turn-on voltage [V]	Max. brightness [cd/m^2^]	Power eff. at Max. brightness [lm/W]
ZnO	10.5	25,900 @ 28.4 V	72.9	7.9	15,620 @ 26.8 V	1.2
P(VDF-TrFE-CFE)	29.1	6,030 @ 108.9 V	25.4	63.3	<100	<0.1
AC-OLED	9.0	13,940 @ 23.8 V	50.3	6.7	20,130 @ 19.7 V	1.7
