# Supplementary material for: Achieving High Performance in AC-Field Driven Organic Light Sources
Source: Sci Rep. 2016 Apr 11;6:24116. doi: 10.1038/srep24116 (PMC4827088; doi:10.1038/srep24116)
Supplement: Supplementary Information [file srep24116-s1.pdf]

Supporting Information

Achieving High Performance in AC-Field Driven Organic Light Sources

Junwei Xu[1], David L. Carroll[1, *], Gregory M. Smith[1], Chaochao Dun[1], and Yue Cui[1,2]

[1]Center for Nanotechnology and Molecular Materials, and Department of Physics, Wake Forest University, Winston-Salem, NC 27109, U.S.A.
[2]Key Laboratory of Luminescence and Optical Information (Ministry of Education), Institute of Optoelectronics Technology, Beijing Jiaotong University, Beijing 100044, P. R. China.
[*] E-mail: *carroldl@wfu.edu*

In this work we have tacitly taken the proposed mechanisms of the main body of literature (as listed below)[1-24] as our perspective of how these devices operate. Specifically, we suggest that local polarization, associated with a defect or interface, is responsible in some way, as the origin of charge within the device. That the polarization current due to dE/dt, is proportional to the creation of polarons at the interfaces and defects of the emitter and is thus related to what we have termed "polarization-induced current," is clear from the literature cited. Importantly, this polarization-induced current has the same field dependence and behavior as that of a simple polarization current. This we believe to be well discussed in the references provided.

In this work, however, we suggest that such mechanisms can leave behind transient and uncompensated charge, particularly for the cases of the double insulating or the single layer insulator, AC-field activated device. We further suggest that the "gate-like" semiconductor layer proposed can provide for compensation of this charge by allowing direct injection under ½ of the power cycle, while still allowing for the operation of a "field-activated" device on the other ½ of the power cycle. We emphasize that however one sees the exact mechanism of polaron and exciton formation, it is clear that our results, based upon charge compensation with the device during one power cycle, has led to a significant increase in performance brightness and efficiency. So while we believe the standing model of AC field activated organic light emitting devices based on induced polarization-based currents is correct, our results in this work are not tied to its exact details.

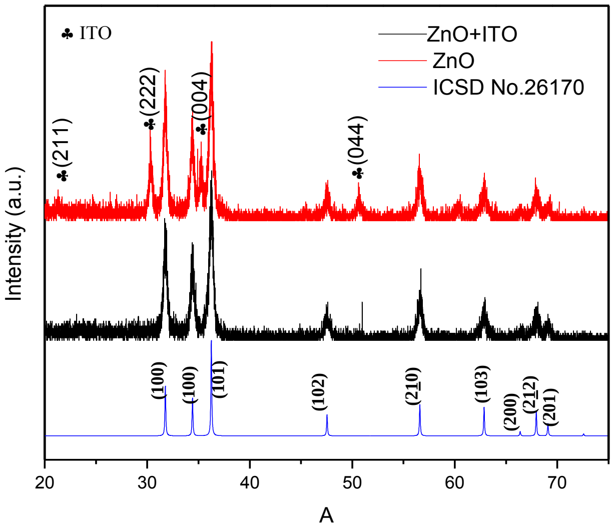

SI 1 | XRD patterns of ZnO nanoparticles on SiO_2 substrate and ITO coated glass.

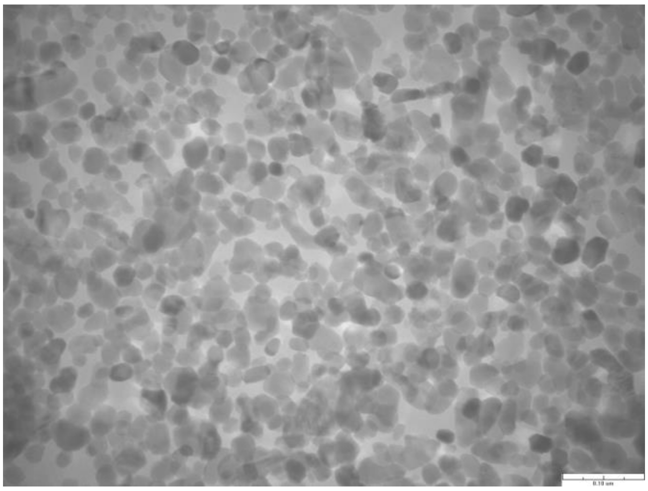

SI 2 | TEM images of ZnO nanoparticles (average diameter ~ 35nm).

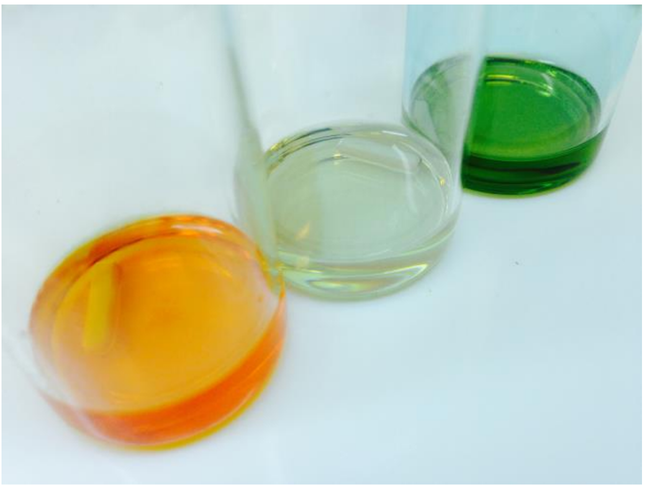

SI 3 | Solutions F4TCNQ (orange), of Poly-TPD (transparent), and Poly-TPD:F4TCNQ (green) in weight ratio of 10:1.

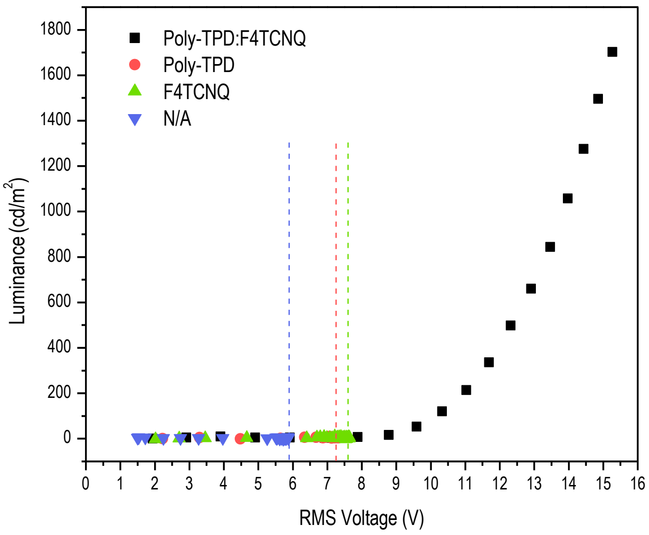

SI 4 | Luminance – RMS voltage characteristic of the AC-OEL devices employing Poly-TPD:F4TCNQ, Poly TPD, and F4TCNQ as HGL and AC-OLED.

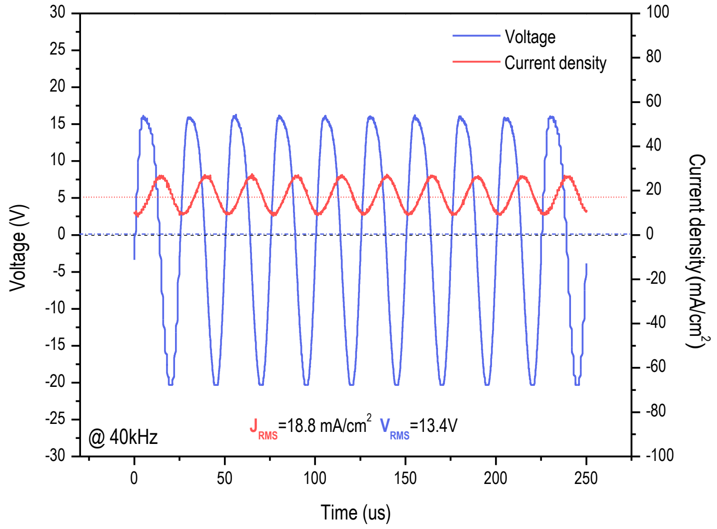

(a)

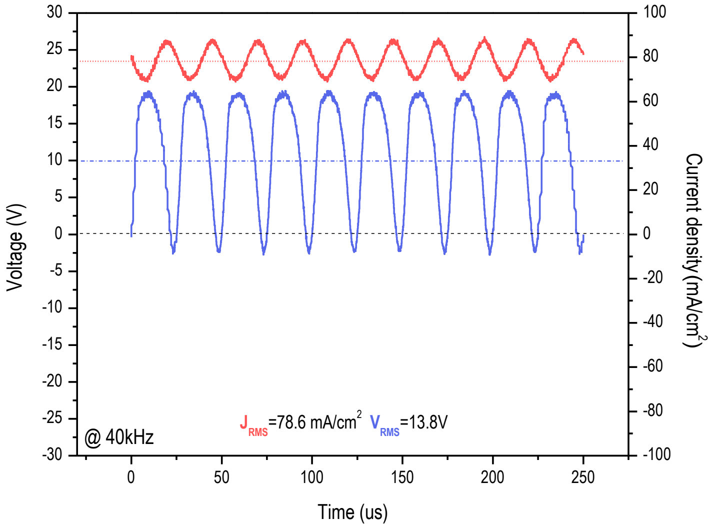

(b)

SI 5 | The voltage waveform and current waveform at 40kHz before without DC voltage offset (a) and with a DC offset (b) of ZnO based AC-OEL device.

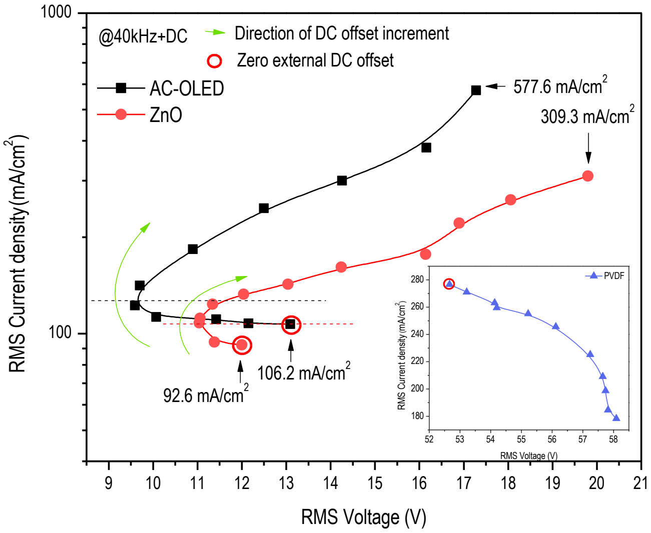

SI 6 | Electric response of additional DC voltage offset loading with AC driving voltage in the AC-OEL devices. RMS current density versus RMS voltage characteristics of the AC-OEL devices with ZnO gate, P(VDF-TrFE-CFE) insulator and AC-OLED while an increasing DC voltage offset are added to AC driving voltage.

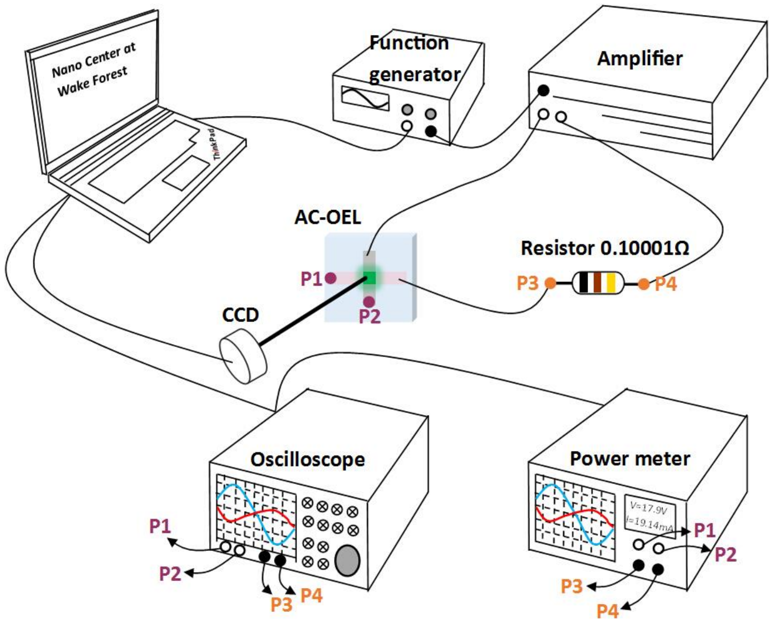

SI 7 | Luminescent and electric characteristics test system for AC-OEL devices.

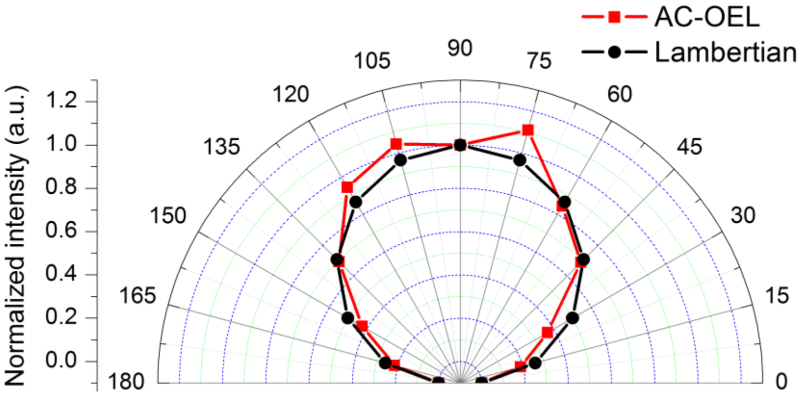

SI 8 | Lambertian patterns of actual emission and ideal panel emission.

ZnO is a common semiconductor with a high-refractive-index (n=2.4) compared with that of organic polymers and glass. It is a concern that multiple reflections occur at the top and bottom surfaces of ZnO layers. If the intensities of reflected beams can destructively interfere and cancel each other, all the energy of the beam must be in the transmitted ray. However, anti-reflective structure requires glass substrate a higher refractive-index than ZnO layer. The refractive-index of glass substrate used in this work is about 1.5, which implies ZnO layer cannot be used as an optical out-coupler no matter how thick it is. In fact, ZnO layer obstructs light out-coupling from the devices because of possible total internal reflection at the interface between organic layer and ZnO layer.

A DC offset was added sine driving voltage to facilitate the electron extraction over the ZnO gate. **SI 5** (a) and (b) show the current and voltage waveforms with and without a DC voltage offset (0.4V) respectively. The J_{RMS} of device increases dramatically from 18.8 mA/cm^2 to 78.6 mA/cm^2, which is attributed to enhanced electron extraction rate via ZnO gate.

SI 6 shows J_{RMS} - V_{RMS} characteristic in the devices with ZnO gate, PVDF insulator or AC-OLED. Considering the mechanisms of charge generation in Poly-TPD:F4TCNQ under intense high-frequency electric field, a great number of electrons and holes are created and accumulated in high-frequency AC cycles. Loading an external DC voltage offset, the massive electrons in Poly-TPD:F4TCNQ are drifted away to ITO over ZnO gate layer and leave empty sites as positive carriers. Promoted holes generation in the Poly-TPD:F4TCNQ suggests stronger hole injection to EML (PVK:Ir(ppy)$_3$), leading to a brighter device. With increase of electric field by promoting DC offset in AC driving voltage, the current density across the device increases rapidly. A promoted current injection with the increasing DC offset is observed in the device without gate or insulator since a uniform electric field is still capable to affect electron extraction in the absence of gate or insulator. The inset of **SI 6** shows the direct current injection is significant suppressed, which suggests that the capacitor property of the AC-OEL device due to high k insulator has dominant influence. So it is not hard to understand L - V_{RMS} curve of PVDF insulator device (the inset in **Figure 5** (h)).

Tunneling model:[26,27]

$$J \propto V^2 \exp\left(\frac{-\kappa}{V}\right) \tag{1}$$

$$\kappa = \frac{8\pi\sqrt{2m^*}\varphi^{3/2}}{3qh}$$ for a triangular barrier

m*:the effective mass

φ:the barrier height

P-N junction model: [28]

$$J = \left(\frac{9\pi}{8}\right)^{1/2} \varepsilon_o \varepsilon_r \left[\frac{2q\mu_p\mu_n(\mu_p+\mu_n)}{\varepsilon_o\varepsilon_r B}\right]^{1/2} \frac{V^2}{L^3} \quad (2)$$

The standard power efficiency calculation for AC driven OEL devices has been given by (nearly lambertian emission shown as **SI 8**),[29]

$$\eta_p[\mathrm{lm/W}] = \pi \cdot \frac{L[\mathrm{cd/m^2}]}{P[\mathrm{W/m^2}]} = \pi \cdot \frac{L[\mathrm{cd/m^2}]}{V_{RMS} \cdot I_{RMS} \cdot \cos\theta / \mathrm{A}}$$

where L is luminance, θ is the phase angle between sinusoidal voltage and current, and A is active area of an EL pixel.

References:

1. Fengling, Z. et al. AC Electroluminescence From Single Layer Organic Thinfilm Electroluminescent Devices Made Of Polymer Ppv. Inf. Display, 1997., Proc. Fourth Asian Symp. 85–86 (1997). doi:10.1109/ASID.1997.631405

2. Lee, S.-B., Fujita, K. & Tsutsui, T. Emission Mechanism of Double-Insulating Organic Electroluminescence Device Driven at AC Voltage. Jpn. J. Appl. Phys. 44, 6607 (2005).

3. Sung, J. et al. AC field-induced polymer electroluminescence with single wall carbon nanotubes. Nano Lett. 11, 966–72 (2011).

4. Cho, S. H. et al. High performance AC electroluminescence from colloidal quantum dot hybrids. Adv. Mater. 24, 4540–6 (2012).

5. Perumal, A. et al. Novel Approach for Alternating Current (AC)-Driven Organic Light-Emitting Devices. Adv. Funct. Mater. 22, 210–217 (2012).

6. Perumal, A., Lüssem, B. & Leo, K. Ultra-bright alternating current organic electroluminescence. Org. Electron. physics, Mater. Appl. 13, 1589–1593 (2012).

7. Chen, Y. et al. Effect of multi-walled carbon nanotubes on electron injection and charge generation in AC field-induced polymer electroluminescence. Org. Electron. 14, 8–18 (2013).

8. Chen, Y. et al. Emission characteristics in solution-processed asymmetric white alternating current field-induced polymer electroluminescent devices. Appl. Phys. Lett. 102, 013307 (2013).

9. Xia, Y. et al. Effects of electrode modification using calcium on the performance of alternating current field-induced polymer electroluminescent devices. Appl. Phys. Lett. 102, 1–5 (2013).

10. Zhao, Y. et al. AC-driven, color- and brightness-tunable organic light-emitting diodes constructed from an electron only device. Org. Electron. 14, 3195–3200 (2013).

11. Chen, Y., Xia, Y., Smith, G. M. & Carroll, D. L. Frequency-Dependent, Alternating Current-Driven, Field-Induced Polymer Electroluminescent Devices with High Power Efficiency. Adv. Mater. 26, 8133–8140 (2014).

12. Chen, Y. et al. High-color-quality white emission in AC-driven field-induced polymer electroluminescent devices. Org. Electron. 15, 182–188 (2014).

13. Chen, Y. et al. Solution-Processable Hole-Generation Layer and Electron-Transporting Layer: Towards High-Performance, Alternating-Current-Driven, Field-Induced Polymer Electroluminescent Devices. Adv. Funct. Mater. 24, 2677–1688 (2014).

14. Chen, Y. et al. Solution-Processed Highly Efficient Alternating Current-Driven Field-Induced Polymer Electroluminescent Devices Employing High- k Relaxor Ferroelectric Polymer Dielectric. Adv. Funct. Mater. 24, 1501–1508 (2014).

15. Fröbel, M., Hofmann, S., Leo, K. & Gather, M. C. Optimizing the internal electric field distribution of alternating current driven organic light-emitting devices for a reduced operating voltage. Appl. Phys. Lett. 104, 071105 (2014).

16. Liu, S.-Y., Chang, J.-H., -Wen Wu, I. & Wu, C.-I. Alternating Current Driven Organic Light Emitting Diodes Using Lithium Fluoride Insulating Layers. Sci. Rep. 4, 7559 (2014).

17. Nie, W. et al. Nano graphite platelets enhanced blue emission in alternating current field induced polymer based electroluminescence devices using Poly (9,9-dioctylfluorenyl-2,7-diyl) as the emitter. Org. Electron. 15, 99–104 (2014).

18. Zhang, L., Nakanotani, H., Yoshida, K. & Adachi, C. Analysis of alternating current driven electroluminescence in organic light emitting diodes: A comparative study. Org. Electron. 15, 1815–1821 (2014).

19. Fröbel, M. et al. White light emission from alternating current organic light-emitting devices using high frequency color-mixing. Phys. Status Solidi 210, 2439–2444 (2013).

20. Fröbel, M. et al. Get it white: color-tunable AC/DC OLEDs. Light Sci. Appl. 4, e247 (2015).

21. Zhao, W., Yang, Z., Jiao, B. & Wu, Z. Organic alternating current electroluminescence device based on 4,4′-bis(N-phenyl-1-naphthylamino) biphenyl/1,4,5,8,9,11-hexaazatriphenylene charge generation unit. Org. Electron. 17, 44–50 (2015).

22. Xu, J. et al. Layered , Nanonetwork Composite Cathodes for Flexible , High-Effi ciency , Organic Light Emitting Devices. Adv. Funct. Mater. 25, 4397–4404 (2015).

23. Xia, Y. et al. High-performance alternating current field-induced chromatic-stable white polymer electroluminescent devices employing a down-conversion layer. J. Lumin. 161, 82–86 (2015).

24. Xia, Y. et al. Alternating current-driven , white field-induced polymer electroluminescent devices with high power efficiency. Org. Electron. 15, 3282–3291 (2014).

25. Micelles, C. et al. Extremely Bright Full Color Alternating Current Electroluminescence of Solution-Blended Fluorescent Polymers with Self-Assembled Block. ACS Nano 7, 10809–10817 (2013).

26. Parker, I. D. Carrier tunneling and device characteristics in polymer light-emitting diodes. J. Appl. Phys. 75, (1994).

27. Braun, D. & Heeger, A. J. Visible light emission from semiconducting polymer diodes. Appl. Phys. Lett. 58, (1991).

28. Blom, P. W. M. & Jong, M. J. M. De. Electrical characterization of polymer light-emitting diodes. IEEE J. Sel. Top. Quantum Electron. 4, 105–112 (1998).

29. Perumal, A. et al. Novel Approach for Alternating Current (AC)-Driven Organic Light-Emitting Devices. Adv. Funct. Mater. 22, 210–217 (2012).
